# Which sample type is better for Xpert MTB/RIF to diagnose adult and pediatric pulmonary tuberculosis?

**DOI:** 10.1042/BSR20200308

**Published:** 2020-08-04

**Authors:** Mengyuan Lyu, Jian Zhou, Yuhui Cheng, Weelic Chong, Kang Wu, Teng Fang, Tianbo Fu, Binwu Ying

**Affiliations:** 1 Department of Laboratory Medicine, West China Hospital, Sichuan University, Chengdu 610041, China; 2 West China Medical School/West China Hospital, Sichuan University, Chengdu, Sichuan Province 610041, P.R. China; 3 Sidney Kimmel School of Medicine, Thomas Jefferson University, Philadelphia, PA, U.S.A.

**Keywords:** diagnostic performance, pulmonary tuberculosis, sample types, Xpert MTB/RIF

## Abstract

**Objective:** This review aimed to identify proper respiratory-related sample types for adult and pediatric pulmonary tuberculosis (PTB), respectively, by comparing performance of Xpert MTB/RIF when using bronchoalveolar lavage (BAL), induced sputum (IS), expectorated sputum (ES), nasopharyngeal aspirates (NPAs), and gastric aspiration (GA) as sample.

**Methods:** Articles were searched in Web of Science, PubMed, and Ovid from inception up to 29 June 2020. Pooled sensitivity and specificity were calculated, each with a 95% confidence interval (CI). Quality assessment and heterogeneity evaluation across included studies were performed.

**Results:** A total of 50 articles were included. The respective sensitivity and specificity were 87% (95% CI: 0.84–0.89), 91% (95% CI: 0.90–0.92) and 95% (95% CI: 0.93–0.97) in the adult BAL group; 90% (95% CI: 0.88–0.91), 98% (95% CI: 0.97–0.98) and 97% (95% CI: 0.95–0.99) in the adult ES group; 86% (95% CI: 0.84–0.89) and 97% (95% CI: 0.96–0.98) in the adult IS group. Xpert MTB/RIF showed the sensitivity and specificity of 14% (95% CI: 0.10–0.19) and 99% (95% CI: 0.97–1.00) in the pediatric ES group; 80% (95% CI: 0.72–0.87) and 94% (95% CI: 0.92–0.95) in the pediatric GA group; 67% (95% CI: 0.62–0.72) and 99% (95% CI: 0.98–0.99) in the pediatric IS group; and 54% (95% CI: 0.43–0.64) and 99% (95% CI: 0.97–0.99) in the pediatric NPA group. The heterogeneity across included studies was deemed acceptable.

**Conclusion**: Considering diagnostic accuracy, cost and sampling process, ES was a better choice than other sample types for diagnosing adult PTB, especially HIV-associated PTB. GA might be more suitable than other sample types for diagnosing pediatric PTB. The actual choice of sample types should also consider the needs of specific situations.

## Introduction

Globally, there were 10 million new tuberculosis (TB) cases and 1.24 million deaths in 2018 alone [1]. A body of studies have confirmed that early diagnosis and treatment can prevent most TB deaths [2–4], and thus excellent diagnostic tools need to be developed. The Xpert MTB/RIF (Cepheid, Sunnyvale, CA, United States) was endorsed as a diagnostic test for use in TB endemic countries by World Health Organization (WHO) in 2010 [5]. A systematic review of Xpert MTB/RIF studies has reported different sensitivities of Xpert MTB/RIF, ranging from 25 to 100% [6]. A study has shown the sensitivity of Xpert MTB/RIF varies with the sample types, samples quality, and bacterial load of samples [7]. Thus, choosing proper sample types is critical to improve the diagnostic performance of Xpert MTB/RIF. Given the high prevalence of pulmonary TB (PTB) [8], we paid attention to the selection of respiratory-related sample types to better diagnose PTB.

The application of urine and stool for diagnosing PTB have been reported [9,10], however, considering high TB burden areas which are usually economically underdeveloped, high cost of Xpert MTB/RIF and relatively low detection rate in non-respiratory samples [11], respiratory-related sample become the first choice for detecting *Mycobacterium tuberculosis* (MTB) in clinical practice, at least for now. Expectorated sputum (ES), induced sputum (IS), bronchoalveolar lavage (BAL) fluid, gastric aspiration (GA), and nasopharyngeal aspirates (NPAs) are considered suitable samples to detect PTB and are regularly collected in clinical practice. ES is readily available, however, has low bacterial load. IS, sputum induced by the inhalation of hypertonic saline, usually has large sample volume and higher quality than ES [12]. BAL has been widely accepted as the most specific sample type used for accurate and timely diagnosis of lung diseases [13,14]. The WHO supports the collection of BAL to diagnose PTB when possible [15]. However, high cost, invasive sampling and the potential risk of hemorrhage, pneumothorax, laryngospasm and other adverse reactions limit its application [16]. It is also worth noting that in clinical practice, bronchoscopy and lavage are not considered as feasible in young children due to the potential risk for anesthesia and need of specialized procedural expertise, unless otherwise specified [17]. NPA is sampled after stimulation of the cough reflex via inserting a small feeding catheter into the nasopharynx [18]. Low operational requirements, less invasive sampling, and ready access enable NPA to become an alternative to BAL for pediatric PTB, but not for adult PTB due to significantly different airway microbial composition between NPA and BAL [17]. Young children, particularly who under 5 years, are unable to expectorate sputum and always swallow sputum in their stomach by mistake, thus GA is collected by a nasogastric tube during night in three consecutive mornings to detect MTB for pediatric PTB [19,20]. Nonetheless, MTB is less likely to be detected in GA than smear in adults, and therefore, and thus GA is not regarded as an option for detecting MTB for adult PTB [21]. Enough volume and consecutive samples of GA support repeat tests to improve the rate of detection [22].

Although there are many samples types to choose from, different samples vary greatly in cost, requirement for operators and operating environment, the degree of sampling invasiveness, quality, volume, and others. It is hard to assess which is the best choice for diagnosing PTB. In addition, few studies pay attention to assess the impact of different sample types on the performance of Xpert MTB/RIF when diagnosing PTB. Therefore, we undertook this systematic review and meta-analysis to compare the detection capacity of Xpert MTB/RIF when using ES, IS, and BAL as samples for diagnosing adult PTB, as well as ES, IS, GA, and NPA for diagnosing pediatric PTB. We aimed to summarize and curate reliable evidence for choosing the proper sample types to diagnose PTB patients at different ages.

## Materials and methods

### Search strategy

We conducted a full search in Web of Science, PubMed, and Ovid on 29 June 2020, with the following terms: ‘tuberculosis’, ‘*mycobacterium tuberculosis’*, ‘TB’, ‘MTB’ AND ‘GeneXpert’, ‘Xpert’, ‘Xpert MTB/RIF’, ‘GX’ AND ‘gastric aspirate’, ‘gastric aspiration’, ‘gastric specimen’, ‘gastric lavage aspiration’, ‘GA’, ‘GLA’, ‘GS’, ‘bronchoalveolar lavage’, ‘bronchoalveolar lavage fluid’, ‘bronchoalveolar washing’, ‘BAL’, ‘BALF’, ‘BW’, ‘induced sputum’, ‘IS’, ‘expectorated sputum’, ‘ES’, ‘nasopharyngeal aspirates’, ‘NPA’. Potential studies were manually identified from reference lists of relevant articles. Any disagreements would be discussed by two independent authors and, if necessary, a third reviewer would arbitrate.

### Inclusion and exclusion criteria

Included studies needed to: (i) be cross-sectional studies, cohort studies, or randomized controlled trials; (ii) focus on PTB patients with or without other diseases; (iii) use two diagnostic methods—Xpert MTB/RIF and culture, and take culture as the reference standard; (iv) collect GA, NPA from children, or BAL from adults, or IS or ES from both; (v) give clear information that participants were adults or children. Exclusion criteria were: (i) reviews, case reports, letters, conference abstracts, and animal experiments; (ii) articles not written in English; (iii) articles with incomplete basic data.

### Quality assessment

Two independent reviewers assessed the quality of included studies according to the Revised Tool for Quality Assessment of Diagnostic Accuracy Studies (QUADAS)-2 [23]. Both risk of bias and applicability concerns would be evaluated as ‘high’, ‘unclear’ or ‘low’. Advice of a third reviewer would be referred if necessary.

### Data extraction and management

All data were collected and entered into Microsoft Excel version 2016. The basic information mainly comprised the first author, publication year, study population’s characteristics, and so on. Diagnostic information included true-positive, false-positive, true-negative, and false-negative. If necessary, we contacted the authors for more essential information, otherwise the trials with incomplete information would be excluded.

### Heterogeneity

The threshold effect and non-threshold effect could result in heterogeneity. The heterogeneity caused by the threshold effect could be explored by performing the Spearman correlation coefficient or plotting summary receiver operating characteristic curves (sROCs). The threshold effect would exist if *P*-value of the Spearman correlation coefficient was less than 0.05 or the points in the plots had a curvilinear (shoulder arm) pattern. If Chi-square *P*-values were less than 0.10, heterogeneity might be caused by a non-threshold effect and a random-effects model would be chosen.

### Sensitivity analysis

Sensitivity analysis was performed to determine the robustness of any treatment effect by removing low-quality studies.

### Statistical analysis

The diagnostic performance of Xpert MTB/RIF was assessed by calculating the pooled sensitivity, specificity, and area under the curve (AUC), with a 95% confidence interval (CI). The forest plots and sROC were also plotted. We performed statistical analysis with Meta-DiSc 1.4 (XI Cochrane Colloquium, Barcelona, Spain) and Review Manager V.5.3 (The Cochrane Collaboration, Software Update, Oxford, U.K.).

## Results

### Studies’ characteristics

We identified 50 articles: 17 [7,24–39] in the adult BAL group, 14 [35,40–52] in the adult ES group, 7 [35,45,50,53–56] in the adult IS group, 2 [57,58] in the pediatric ES group, 6 [57,59–63] in the pediatric GA group, and 9 [59,64–71] in the pediatric IS group from 1791 studies for the meta-analysis. A total of 2864 BAL samples, 18176 ES samples, and 3876 IS samples were collected from adults. A total of 593 ES samples, 1595 GA samples, 2759 IS samples, and 780 NPA samples were collected from children. More than 80% of all samples were preprocessed, including neutralization, centrifugation, or other processing. The volume of BAL, ES, and IS were 1.0–5 ml, ≥1.0 ml, and 0.5–2.0 ml, respectively. The volume of GA varied from 0.1 ml to more than 5 ml, while 0.5–1.5 ml IS was collected from children.

**Figure 1:**
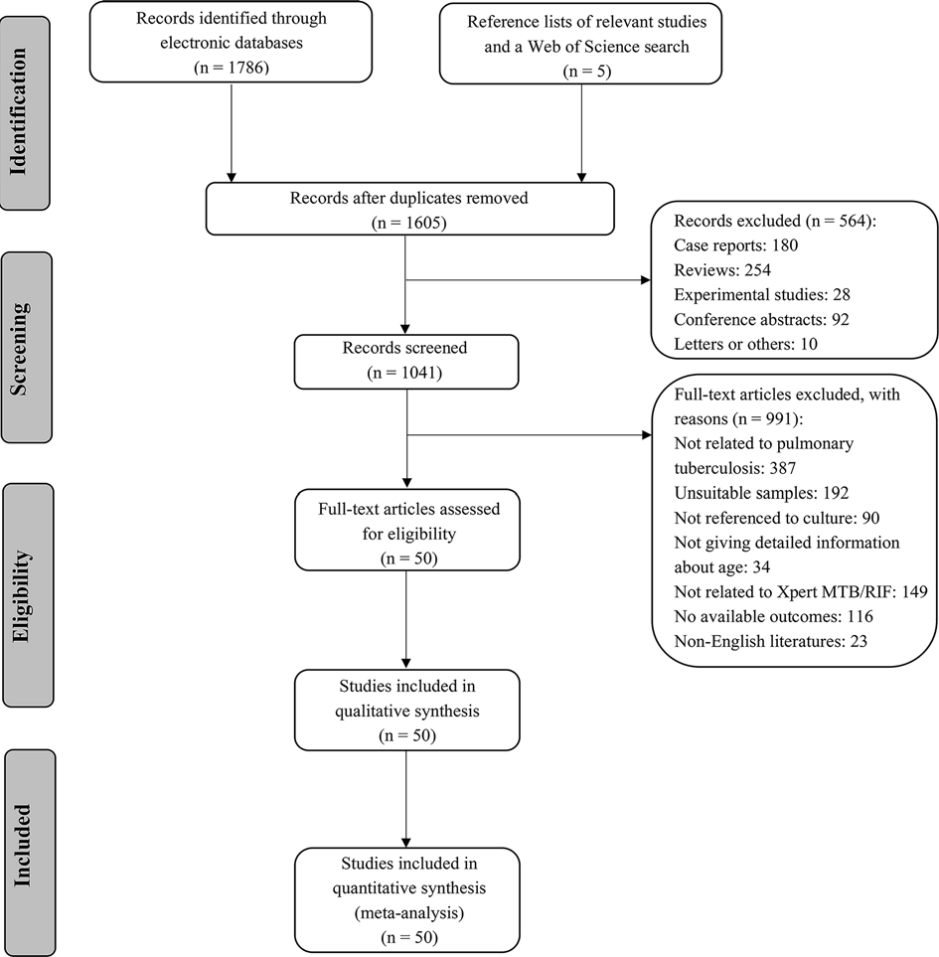
The flow diagram of articles included in this meta-analysis

The flow diagram of articles included in this meta-analysis is presented in Figure 1. The main characteristics of 43 included articles are summarized in Table 1.

**Table 1:** The main characteristics of included trials

Study (year)	Country or Area	Patient recruitment	Sample size	Sample processing	Smear- positive rate (%)	HIV- positive rate (%)	Culture type
**Adult BAL^1^ group**							
Barnard, 2015	South Africa	NA^2^	112	Decontamination and centrifugation	15	NA	MGIT
Gowda, 2018	India	NA	60	Unprocessed	12	7	MGIT
Jo, 2016	South Korea	NA	320	Centrifugation	8	0	MGIT and Ogawa
Khalil, 2015	Pakistan	NA	93	NA	37	NA	LJ^3^
Kilaru, 2019	India	Inpatients and outpatients	56	Decontamination and centrifugation	38	0	MGIT
Ko, 2016	Korea	NA	249	NA	NA	0	MGIT and Ogawa
Lee, 2013	Korea	NA	132	Decontamination and centrifugation	5	0	MGIT and Ogawa
Lu, 2018	China	NA	238	Decontamination	9	NA	MGIT and LJ
Mok, 2016	Singapore	Inpatients and outpatients	158	NA	6	NA	MGIT
Pan, 2018	China	NA	190	Decontamination	19	NA	MGIT
Prakash, 2018	Six countries	Inpatients and outpatients	383	NA	6	NA	MGIT and LJ
Sánchez-Cabral, 2020	Brazil	NA	54	NA	NA	41	MGIT and LJ
Sharma, 2015*	India	NA	131	Decontamination and centrifugation	NA	NA	MGIT and LJ
Theron, 2013	South Africa	NA	154	Centrifugation	10	30	MGIT
To, 2018	China	NA	237	NA	NA	2	NA
Ullah, 2017	Pakistan	NA	98	Decontamination	0	100	NA
Silva, 2019	Brazil	NA	199	NA	NA	NA	MGIT and LJ
**Adult ES^4^ group**							
Al-Darraji, 2013	Malaysia	Prison	125	Decontamination and centrifugation	NA	100	MGIT
Boum, 2016	Uganda	NA	1016	NA	20	66	MGIT
Friedrich, 2011	South Africa	NA	292	Decontamination and centrifugation	97	42	MGIT
Geleta, 2015	Somali	NA	227	Decontamination	26	NA	MGIT and LJ
LaCourse, 2016	Kenya	NA	288	Decontamination	NA	100	MGIT
Luetkemeyer, 2016*	American, Brazil, and Rio de Janeiro	Inpatients and outpatients	992	Unprocessed	15	NA	MGIT and solid media
Madico, 2016	Mbarara and Uganda	Inpatients	261	Decontamination	15	100	MGIT
Pinyopornpanish, 2015	Thailand	NA	57	NA	14	46	MGIT
Rachow, 2011	Tanzania	NA	126	Decontamination	NA	NA	MGIT and LJ
Rasheed, 2019	Pakistan	NA	225	Unprocessed	0	NA	LJ
Rice, 2017*	American	NA	637	Decontamination and centrifugation	30	NA	MGIT and LJ
Sahle, 2019	Ethiopia	Prison	13803	NA	0.1	0.6	MGIT
Sharma, 2015*	India	NA	71	Decontamination and centrifugation	NA	NA	MGIT and LJ
Yeong, 2020	Australia	Inpatients	56	Decontamination	11	0	MGIT
**Adult IS^5^ group**							
Acuna, 2017	Uganda	Inpatients and outpatients	860	Decontamination	89	69	MGIT
Luetkemeyer, 2016*	America and Brazil	NA	324	NA	NA	NA	MGIT and solid media
Mishra, 2020	South Africa	NA	585	Decontamination	12	20	MGIT and LJ
O’Grady, 2012	Zambia	Inpatients	881	Centrifugation	18	71	MGIT
Rice, 2017*	America	NA	653	Decontamination and centrifugation	NA	NA	MGIT and LJ
Sharma, 2015*	India	NA	71	Decontamination and centrifugation	NA	NA	MGIT and LJ
Sohn, 2014	Canada	NA	502	Decontamination	3	2	MGIT
**Pediatric ES group**							
Bates, 2013*	Zambia	Inpatients	142	Decontamination and centrifugation	7	31	MGIT
Reither, 2015	Tanzania and Uganda	NA	451	Decontamination	NA	44	MGIT and LJ
**Pediatric GA^6^ group**							
Bates, 2013*	Zambia	Inpatients	788	Decontamination and centrifugation	4	30	MGIT
Das, 2018*	India and adjoining areas of Nepal	Inpatients and outpatients	106	NA	NA	NA	LJ
Hasan, 2017	Pakistan	NA	49	Decontamination and centrifugation	NA	NA	MGIT
Myo, 2018	Myanmar	NA	231	Decontamination and centrifugation	5	13	LJ
Pang, 2014	China	NA	211	Decontamination and centrifugation	0	NA	MGIT
Sharma 2020	India	Outpatients	210	Decontamination	7	NA	MGIT
**Pediatric IS group**							
Das, 2018*	India and adjoining areas of Nepal	Inpatients and outpatients	8	Nebulization	NA	NA	LJ
LaCourse, 2014	Malawi	NA	300	NA	0	17	MGIT
Nicol, 2011	South Africa	NA	452	Decontamination and centrifugation	6	24	MGIT
Nicol, 2013	South Africa	NA	115	Decontamination and centrifugation	3	15	MGIT
Nicol, 2018	South Africa	NA	367	Decontamination and centrifugation	NA	19	MGIT and LJ
Sekadde, 2013	Uganda	Mixed	250	Digestion and decontamination	6	42	MGIT and LJ
Togun, 2015	Gambia	Outpatients	487	Digestion, decontamination, and centrifugation	1	0	MGIT and LJ
Zar, 2012*	South Africa	Inpatients	396	Decontamination and centrifugation	5	20	MGIT
Zar, 2013*	South Africa	NA	384	Decontamination and centrifugation	1	8	MGIT
**Pediatric NPA^7^ group**							
Zar, 2012*	South Africa	Inpatients	396	Decontamination and centrifugation	4	20	MGIT
Zar, 2013*	South Africa	NA	384	Decontamination and centrifugation	NA	NA	MGIT

* These studies were divided into at least two groups.

a, BAL. b, Not available. c, Löwenstein–Jensen medium. d, ES. e, IS. f, GA. g, NPAs.

### Risk of bias

The risk of bias of the included studies is shown in Table 2.

**Table 2:** The risk of bias of the included studies

Study	Risk of bias				Applicability concerns		
	Patient selection	Index test	Reference standard	Flow and timing	Patient selection	Index test	Reference standard
**Adult BAL^1^ group**							
Barnard, 2015	unclear	unclear	low	low	low	low	low
Gowda, 2018	unclear	low	low	low	low	low	low
Jo, 2016	unclear	low	low	low	unclear	low	low
Khalil, 2015	unclear	unclear	unclear	unclear	low	low	low
Kilaru, 2019	low	low	low	low	low	low	low
Ko, 2016	unclear	unclear	unclear	unclear	low	low	low
Lee, 2013	unclear	low	low	low	low	low	low
Lu, 2018	low	low	low	low	low	low	low
Mok, 2016	unclear	low	low	low	low	low	low
Pan, 2018	unclear	low	low	low	low	low	low
Prakash, 2018	low	low	low	low	low	low	high
Sánchez-Cabral, 2020	low	low	low	low	low	low	low
Sharma, 2015*	low	low	low	low	low	low	low
Silva, 2019	unclear	unclear	low	low	low	low	low
Theron, 2013	unclear	low	low	low	low	low	low
To, 2018	unclear	low	low	low	unclear	low	low
Ullah, 2017	unclear	low	low	low	high	low	low
**Adult ES^2^ group**							
Al-Darraji, 2013	high	low	low	low	low	low	unclear
Boum, 2016	low	low	low	low	low	low	low
Friedrich, 2011	unclear	low	low	low	low	low	low
Geleta, 2015	low	low	low	low	low	low	low
LaCourse, 2016	high	low	low	low	low	low	low
Luetkemeyer, 2016*	low	low	low	low	low	low	low
Madico, 2016	high	low	low	low	low	low	low
Pinyopornpanish, 2015	low	low	low	low	low	low	low
Rachow, 2011	low	low	low	low	low	low	low
Rasheed, 2019	low	low	low	low	low	low	low
Rice, 2017*	low	low	low	low	low	low	low
Sahle, 2019	low	low	low	low	low	low	unclear
Sharma, 2015*	low	low	low	low	low	low	low
Yeong, 2020	low	low	low	low	low	low	low
**Adult IS^3^ group**							
Acuna, 2017	high	low	low	low	low	low	low
Luetkemeyer, 2016*	unclear	low	unclear	low	low	low	low
Mishra, 2020	low	low	low	low	low	low	low
O’Grady, 2012	unclear	low	low	low	low	low	low
Rice, 2017*	unclear	unclear	unclear	low	low	low	low
Sharma, 2015*	low	low	low	low	low	low	low
Sohn, 2014	low	unclear	unclear	low	low	low	low
**Pediatric ES group**							
Bates, 2013*	low	low	low	low	low	low	low
Reither, 2015	low	low	low	low	low	low	low
**Pediatric GA^4^ group**							
Bates, 2013*	unclear	low	low	low	low	low	low
Das, 2018*	unclear	unclear	unclear	low	low	low	low
Hasan, 2017	unclear	low	low	low	low	low	low
Myo, 2018	unclear	low	low	low	low	low	low
Pang, 2014	low	low	low	low	high	low	low
Sharma, 2020	low	low	low	low	low	low	low
**Pediatric IS group**							
Das, 2018*	unclear	unclear	unclear	low	low	low	low
LaCourse, 2014	low	unclear	unclear	low	high	low	low
Nicol, 2011	low	low	low	low	low	low	low
Nicol, 2013	low	unclear	unclear	low	low	low	low
Nicol, 2018	low	low	unclear	low	low	low	low
Sekadde, 2013	high	low	low	low	low	low	low
Togun, 2015	low	low	low	low	high	low	low
Zar, 2012*	low	low	low	low	low	low	low
Zar, 2013*	low	low	low	low	low	low	low
**Pediatric NPA^5^ group**							
Zar, 2012*	low	low	low	low	low	low	low
Zar, 2013*	low	low	low	low	low	low	low

* The studies of these authors were divided into at least two groups.

a, BAL. b, ES. c, IS. d, GA. e, NPAs.

Among 17 studies in the adult BAL group, 5 articles were assessed as low risk and the rest had an unclear risk on the patient selection. For the index test, 13 publications had a low risk and 4 publications had an unclear risk. In the aspect of reference standard and flow and timing, all trials gave clear description about trial details, except for two [27,29]. A total of 14 studies had low concern for applicability, however, the risk of patient selection of 2 studies [37,72] were unclear and that of Ullah et al. [38] was high.

For 14 studies in the adult ES group, 10 studies were assessed as low risk on the patient selection, 3/14 were considered as high risk and the remaining was identified as unclear risk. All of them showed low risk on the index test, reference standard and flow, and timing. Low concern of patient selection and index test appeared in all 14 publications. All studies were evaluated as low risk on reference standard except for two [40,51].

For seven studies in the adult IS group, three articles had low risk of patient selection, three articles had unclear risk of patient selection and only one had high risk of patient selection. Two studies [50,56], which did not state whether the results from two methods were double-blind, assessed as unclear risk on the index test and reference standard. Besides, Luetkemeyer et al. [45] also had an unclear risk on reference standard. All articles in this group were considered as low risk on applicability concerns.

In the pediatric ES group, two studies showed low risk in risk of bias and applicability concerns.

In the pediatric GA group, two articles had low risk of patient selection while others were of unclear risk. Unclear risk of the index test and reference standard only appeared in one publication. Five trials had low risk of flow and timing and applicability concerns, and one [62] was evaluated as high risk for patient selection.

In the pediatric IS group, all studies had low risk of patient selection except two [59,68]. Das et al. [59], LaCourse et al. [64], and Nicol et al. [66] had unclear risk of the index test and reference standard. Five articles had low concern of applicability, while another two studies [64,69] were assessed as high risk of patient selection.

In the pediatric NPA group, two studies showed low risk of bias and applicability concerns.

### Heterogeneity

The value of Spearman correlation coefficient showed that the threshold effects did not exist in the adult BAL group (Spearman correlation coefficient: 0.158; *P*=0.727), adult ES group (Spearman correlation coefficient: 0.251; *P*=0.387), adult IS group (Spearman correlation coefficient: 0.321; *P*=0.482), pediatric GA group (Spearman correlation coefficient: −0.257; *P*=0.623), and pediatric IS group (Spearman correlation coefficient: 0.200; *P*=0.606). Besides, the distribution of points in sROC curve also confirmed this. Due to the limited articles in the pediatric ES group and pediatric NPA group, Spearman correlation coefficient and sROC were unavailable.

**Figure 2:**
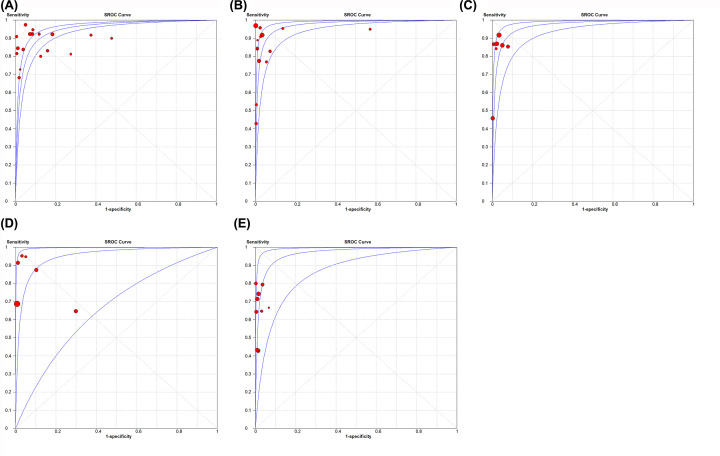
sROC curve for Xpert MTB/RIF of five groups (**A**) sROC of adult BAL group; (**B**) sROC of adult ES group; (**C**) sROC of adult IS group; (**D**) sROC of pediatric GA group; (**E**) sROC of pediatric IS group.

The value of Chi-square *P*-values indicted heterogeneity was caused by non-threshold effects in the adult BAL group (Chi-square: 42.33; *P*<0.001), adult ES group (Chi-square: 52.15; *P*<0.001), adult IS group (Chi-square: 18.95; *P*=0.004), pediatric ES group (Chi-square: 13.89; *P*=0.002) and pediatric GA group (Chi-square: 47.45; *P*<0.001). However, non-threshold effects were not detected in the pediatric IS group (Chi-square: 8.40; *P*=0.396), and pediatric NPA group (Chi-square: 1.57; *P*=0.211). The sROC curves for Xpert MTB/RIF of five groups (adult BAL group, adult ES group, adult IS group, pediatric GA group, and pediatric IS group) are shown in Figure 2.

### Results on diagnostic accuracy

The diagnostic performance of Xpert MTB/RIF was calculated, compared with culture. Xpert MTB/RIF showed the pooled sensitivity, pooled specificity, and AUC were 87% (95% CI: 0.84–0.89), 91% (95% CI: 0.90–0.92) and 95% (95% CI: 0.93–0.97) in the adult BAL group; 90% (95% CI: 0.88–0.91), 98% (95% CI: 0.97–0.98) and 97% (95% CI: 0.95–0.99) in the adult ES group; 86% (95% CI: 0.84–0.89), 97% (95% CI: 0.96–0.98) and 98% (95% CI: 0.94–0.99) in the adult IS group; 80% (95% CI: 0.72–0.87), 94% (95% CI: 0.92–0.95) and 95% (95% CI: 0.87–1.00) in the pediatric GA group; 67% (95% CI: 0.62–0.72), 99% (95% CI: 0.98–0.99) and 97% (95% CI: 0.92–1.00) in the pediatric IS group, respectively. The pooled sensitivity and pooled specificity were 14% (95% CI: 0.10–0.19) and 99% (95% CI: 0.97–1.00) in the pediatric ES group and 54% (95% CI: 0.43–0.64) and 99% (95% CI: 0.97–0.99) in the pediatric NPA group, respectively. Due to the limited articles in the pediatric ES group and pediatric NPA group, the AUC was unavailable.

**Figure 3:**
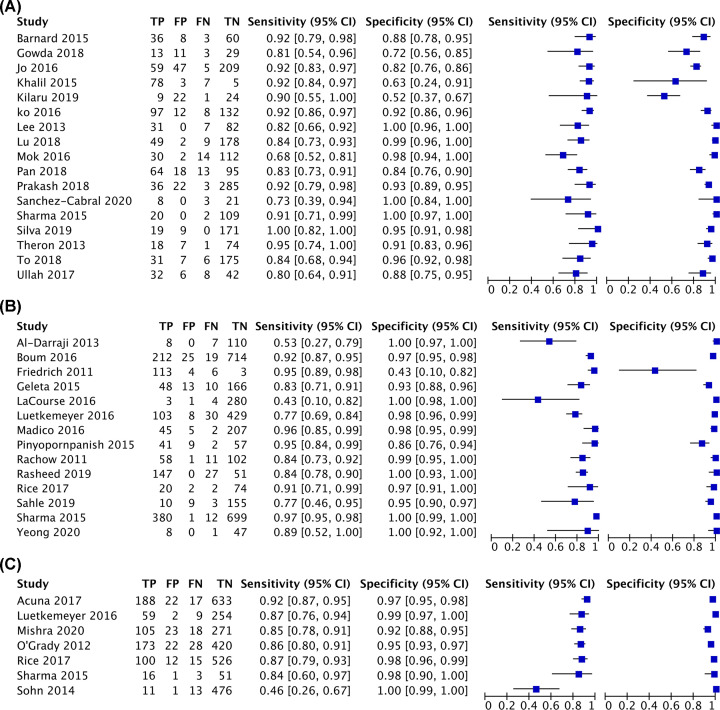
The summary plots of adult PTB (**A**) Summary plots of adult BAL group; (**B**) summary plots of adult ES group; (**C**) summary plots of adult IS group. Abbreviations: FN, false-negative; FP, false-positive; TP, true-positive; TN, true-negative.

**Figure 4:**
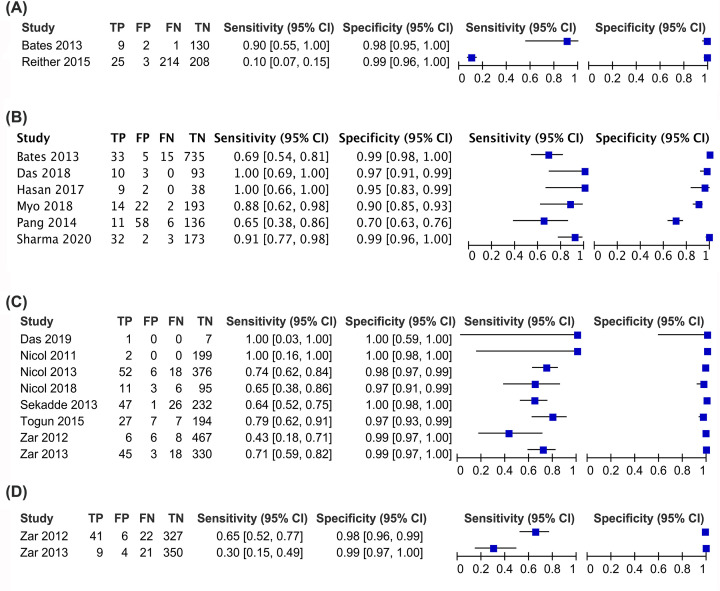
The summary plots of pediatric PTB (**A**) Summary plots of pediatric ES group; (**B**) summary plots of pediatric GA group; summary plots of pediatric IS group; (**D**) summary plots of pediatric NPA group. Abbreviations: FN, false-negative; FP, false-positive; TN, true-negative; TP, true-positive.

The summary plots of Xpert MTB/RIF for adult and children are presented in Figures 3 and 4, respectively.

Subgroup analysis was carried out to determine the potential source of heterogeneity among included articles. The detailed information is presented in Supplementary Table S1.

## Discussion

Since we only included studies using Xpert MTB/RIF, all comparisons made were between the types of biological samples used. For adults, choosing ES as sample provided better diagnostic performance than BAL or IS. For children, GA showed superior diagnostic capacity to ES, IS, or NPA. Of note, GA also has its own disadvantages including invasive sampling and needing approximately three consecutive days for collecting samples. The actual choice needs to be decided according to specific situations.

It is particularly notorious that microbiology methods (including sputum microscopy and culture), immunological methods [interferon-γ release assay (IGRA) and T-cell spot (T-SPOT)] and molecular methods [polymerase chain reaction (PCR)] are widely applied worldwide before the Xpert MTB/RIF came to market. The celerity and convenience of sputum microscopy make it the most commonly used method in clinical practice, nonetheless low sensitivity (20–40%) is the fatal defect of the sputum microscopy [73]. Culture, as the reference standard for the diagnosis of TB, requires too long detection time and a highly equipped laboratory. Thus, culture is greatly limited in some developing regions typically facing a high TB burden. IGRA and T-SPOT are effective ways to screen latent TB infection. However, inoculating Bacille Calmette–Guérin (BCG), prior infection and inhalation of non-pathogenic mycobacterium can result in false positivity of IGRA and T-SPOT [74]. Thus, the clinical value of these two diagnostic methods is somewhat discounted in TB endemic areas. Although PCR is a relatively sensitive method to detect MTB, high requirement of operator skills and a laboratory environment also restrict its application. The emergency of Xpert MTB/RIF meets the urgent demand for rapid, simple, integrated, and fully automated detective methods of MTB detection. A myriad of randomized controlled trials (RCTs) have been carried out to assess the diagnostic performance of Xpert MTB/RIF and found the detection rate of MTB is dramatically improved by this novel molecular tool [75]. Apparent benefit brought by Xpert MTB/RIF to PTB patient individually and public health enables this molecular tool to be rapidly accepted and applied all over the world.

However, Xpert MTB/RIF still faces some challenges of inadequate sensitivity when diagnosing smear-negative PTB, pediatric PTB, HIV-associated PTB, and extra-PTB [76,77]. In order to increase diagnostic yield, the Xpert MTB/RIF Ultra (Ultra; Cepheid) appeared. Based on the same platform as the Xpert MTB/RIF, the Ultra harbors some improvements including using two PCR amplification targets (*IS6110* and *IS1081*) and a melt curve analysis [78]. These two changes confer Ultra’s lower limit of detection than Xpert MTB/RIF and culture [78]. The following clinical trials have found that improper sample types and poor sample quality still have an adverse influence on the sensitivity of Ultra [79,80]. Obviously, no matter how sensitive the detection method is, choosing proper sample type and ensuring the quality of sample are crucial.

In our systematic review, choosing ES as sample provided better diagnostic performance (sensitivity, specificity, and AUC) than BAL or IS for adult PTB. Since both BAL and IS had a higher bacterial load than ES [81,82], they should theoretically provide better diagnostic performance than ES. However, our study showed opposite results. One reason is the smaller volume of IS, ranging from 0.5 to 2 ml, whereas the volume of ES ranges from 1 ml to more than 2 ml. This inconsistency may also be explained by the ratio of processed samples. The ratio of processed samples in the adult ES group was higher than that in the adult BAL group. In addition, we also found there was a high agreement in diagnosing adult PTB when using IS and BAL as sample. Considering cost, sampling method, acceptance and other factors, IS might be a better choice than BAL to diagnose PTB. Blau et al. [83] gave the interpretation of this results that sampling from one or two parts of lung led to poor repeatability and limited detection rate when taking BAL as sample to diagnose lung infection diseases. Garcia et al. [84] found the gene transcriptional patterns of MTB in BAL and sputum were similar, and additionally, sputum could reflect the physiological state of MTB in the lower airway of PTB patients. Conde et al. [85] conducted a trial and reconfirmed the higher diagnostic yield of taking IS (relative to BAL) as sample to diagnose PTB, in line with our results.

For pediatric PTB, similar specificity and AUC appeared when using IS and GA as sample, nonetheless, Xpert MTB/RIF showed much higher sensitivity when using GA as sample to diagnose pediatric PTB. Although GA is sampled by invasive ways and need approximately three consecutive days for collecting samples, the characteristics of lower probability of contamination, easy sample collection, and sufficient sample volume still make GA become an ideal sample type to increase detection rate for pediatric PTB. Ruiz Jimenez et al. [86] may agree with our results that GA is an excellent sample for diagnosing PTB, and IS should be considered as a supplementary sample type. However, Zar et al. [87] drew an opposite conclusion by conducting a prospective study, with IS for diagnosing PTB and GA as supplementary. The differences may result from different diagnostic tools and the variation in sample collection. The culture, used in their study [87], was unable to detect dead MTB which was caused by the neutralization of GA [88], while Xpert MTB/RIF used in our included studies can detect both dead and live MTB and is not affected by sample neutralization. Furthermore, sample collection, technician skill level, the time from sampling to testing and choice of pre-processing methods also influence finial accuracy [89]. Thus, it is worthy for us to pay attention to the standardization of GA sampling.

Subgroup analysis showed that for adult PTB, superior diagnostic performance appeared in the smear-positive subgroup whether using BAL, ES, or IS as samples. Xpert MTB/RIF showed similar sensitivity whether using smear-positive BAL, smear-positive ES or smear-positive IS, while the highest AUC (90%) was identified when using BAL to diagnose smear-negative PTB. BAL was sampled directly from the lesion region, which was more suitable and reliable for detecting PTB patients with low bacterial load [90,91]. In addition, outperformed capacity of Xpert MTB/RIF appeared when detecting MTB in IS or ES than in BAL for HIV-positive patients. Singer-Leshinsky et al. [92] obtained consistent results with ours that collecting IS from HIV patients may be more beneficial than others. Sample pre-procession steps, the number of samples, the immune status of patient, and the degree of disease severity are the potential confounding factors. More sound evidence is still lacking to support the value of ES in diagnosing HIV-associated adult PTB, which need to be further confirmed.

For pediatric PTB, we reconfirmed the strong association between smear status, bacterial load, and the diagnostic performance of Xpert MTB/RIF. Besides, testing for MTB in IS from children provided a little additional diagnostic yield from the prospective of specificity and AUC for Xpert MTB/RIF when diagnosing smear-negative PTB, however, Xpert MTB/RIF had higher likelihood to detect MTB in GA from these smear-negative children who really suffered from PTB. Maynard-Smith et al. [6] reported that detecting MTB in GA for sputum-scarce PTB was a good choice. However, Maynard-Smith et al. did not analyze the diagnostic accuracy of Xpert MTB/RIF when detecting MTB in IS from children and make further comparisons. The diagnostic value of GA and IS for smear-negative PTB for children still need to be explored. In the pediatric IS group, subgroup analysis showed that the pooled sensitivity was very heterogeneous between HIV-positive and HIV-negative subgroups and higher sensitivity was in the HIV-positive subgroup. This result was contrary to Connell et al. [93], however, supported by Nicol et al. [65] The smear status, disease severity and samples’ pre-processing may affect the results, and a further research should be warranted.

## Strengths and limitations

Our study made a rigorous and comprehensive analysis and comparison about the diagnostic performance of Xpert MTB/RIF using different sample types for PTB patients with different ages by including as many studies as possible. Furthermore, we performed reasonable subgroup analysis to identify the source of heterogeneity and potential factors. However, the present study also has some limitations. Limited articles in the pediatric ES group and pediatric NPA group restricted us from further analysis. Some factors including cost, the tolerance of patients and sample contamination rate should be taken into consideration for final choice of diagnostics. Most included articles only used one sample type to analyze the performance of Xpert MTB/RIF, and thus the comparison of different sample types may be influenced by a number of potential confounding factors such as patient characteristics and the process of sampling. Thus, more eligible trials are needed.

## Conclusion

When considering diagnostic performance of Xpert MTB/RIF, sampling method and patients’ tolerance, using ES as sample might be a better choice to diagnose adult PTB than using BAL and IS. For pediatric PTB, GA was superior to other samples. However, the invasive sampling and relatively long time of collecting samples also should be considered. The actual choice of sample types needs to be decided according to specific situations.

## Supplementary Material

Supplementary Table S1

## Data Availability

All data generated or analyzed in the present study are contained and presented in this document.

## Abbreviations

AUC: area under the curve
BAL: bronchoalveolar lavage
CI: confidence interval
ES: expectorated sputum
GA: gastric aspiration
IGRA: interferon-γ release assay
IS: induced sputum
MTB: *Mycobacterium tuberculosis*
NPA: nasopharyngeal aspirate
PCR: polymerase chain reaction
PTB: pulmonary tuberculosis
sROC: summary receiver operating characteristic
TB: tuberculosis
T-SPOT: T-cell spot
WHO: World Health Organization

## References

[1] WHO (2019) Global tuberculosis report 2019. https://www.who.int/tb/publications/global_report/en/

[2] Chee C.B.E., Reves R., Zhang Y. and Belknap R. (2018) Latent tuberculosis infection: opportunities and challenges. Respirology 23, 893–900 29901251 10.1111/resp.13346

[3] Lawn S.D., Mwaba P., Bates M., Piatek A., Alexander H., Marais B.J. et al. (2013) Advances in tuberculosis diagnostics: the Xpert MTB/RIF assay and future prospects for a point-of-care test. Lancet Infect. Dis. 13, 349–361 10.1016/S1473-3099(13)70008-2 23531388 PMC4844338

[4] Yew W.W. and Yoshiyama T. (2018) Epidemiological, clinical and mechanistic perspectives of tuberculosis in older people. Respirology 23, 567–575 29607596 10.1111/resp.13303

[5] Walzl G., McNerney R., du Plessis N., Bates M., McHugh T.D., Chegou N.N. et al. (2018) Tuberculosis: advances and challenges in development of new diagnostics and biomarkers. Lancet Infect. Dis. 18, e199–e210 10.1016/S1473-3099(18)30111-7 29580818

[6] Maynard-Smith L., Larke N., Peters J.A. and Lawn S.D. (2014) Diagnostic accuracy of the Xpert MTB/RIF assay for extrapulmonary and pulmonary tuberculosis when testing non-respiratory samples: a systematic review. BMC Infect. Dis. 14, 709 10.1186/s12879-014-0709-7 25599808 PMC4298952

[7] Theron G., Peter J., Meldau R., Khalfey H., Gina P., Matinyena B. et al. (2013) Accuracy and impact of Xpert MTB/RIF for the diagnosis of smear-negative or sputum-scarce tuberculosis using bronchoalveolar lavage fluid. Thorax 68, 1043–1051 10.1136/thoraxjnl-2013-203485 23811536 PMC5523966

[8] Goussard P. and Gie R. (2014) The role of bronchoscopy in the diagnosis and management of pediatric pulmonary tuberculosis. Expert Rev. Respir. Med. 8, 101–109 10.1586/17476348.2013.863712 24378192

[9] Heydari A.A., Movahhede Danesh M.R. and Ghazvini K. (2014) Urine PCR evaluation to diagnose pulmonary tuberculosis. Jundishapur J. Microbiol. 7, e9311 10.5812/jjm.9311 25147688 PMC4138658

[10] Konno A., Narumoto O., Matsui H., Takeda K., Hirano Y., Shinfuku K. et al. (2019) The benefit of stool mycobacterial examination to diagnose pulmonary tuberculosis for adult and elderly patients. J. Clin. Tuberc. Other Mycobact. Dis. 16, 100106 10.1016/j.jctube.2019.100106 31720430 PMC6830132

[11] Walters E., Demers A.M., van der Zalm M.M., Whitelaw A., Palmer M., Bosch C. et al. (2017) Stool culture for diagnosis of pulmonary tuberculosis in children. J. Clin. Microbiol. 55, 3355–3365 10.1128/JCM.00801-17 28904186 PMC5703802

[12] Geldenhuys H.D., Kleynhans W., Buckerfield N., Tameris M., Gonzalez Y., Mahomed H. et al. (2012) Safety and tolerability of sputum induction in adolescents and adults with suspected pulmonary tuberculosis. Eur. J. Clin. Microbiol. Infect. Dis. 31, 529–537 10.1007/s10096-011-1344-5 21796347

[13] Heron M., Grutters J.C., ten Dam-Molenkamp K.M., Hijdra D., van Heugten-Roeling A., Claessen A.M. et al. (2012) Bronchoalveolar lavage cell pattern from healthy human lung. Clin. Exp. Immunol. 167, 523–531 10.1111/j.1365-2249.2011.04529.x 22288596 PMC3374285

[14] Meyer K.C. (2007) Bronchoalveolar lavage as a diagnostic tool. Semin. Respir. Crit. Care Med. 28, 546–560 10.1055/s-2007-991527 17975782

[15] World Health Organization (2013) Laboratory testing for Middle East respiratory syndrome coronavirus;. http://www.who.int/csr/disease/coronavirus_infections/MERS_Lab_recos_16_Sept_2013.pdf?ua=1&ua=1

[16] Liu X., Hou X.F., Gao L., Deng G.F., Zhang M.X., Deng Q.Y. et al. (2018) Indicators for prediction of Mycobacterium tuberculosis positivity detected with bronchoalveolar lavage fluid. Infect. Dis. Poverty 7, 22 10.1186/s40249-018-0403-x 29580276 PMC5868051

[17] Kloepfer K.M., Deschamp A.R., Ross S.E., Peterson-Carmichael S.L., Hemmerich C.M., Rusch D.B. et al. (2018) In children, the microbiota of the nasopharynx and bronchoalveolar lavage fluid are both similar and different. Pediatr. Pulmonol. 53, 475–482 10.1002/ppul.23953 29405661 PMC6542268

[18] Lu A.Z., Shi P., Wang L.B., Qian L.L. and Zhang X.B. (2017) Diagnostic value of nasopharyngeal aspirates in children with lower respiratory tract infections. Chin. Med. J. (Engl.) 130, 647–651 10.4103/0366-6999.201595 28303845 PMC5358412

[19] Maciel E.L., Brotto L.D., Sales C.M., Zandonade E. and Sant’anna C.C. (2010) Gastric lavage in the diagnosis of pulmonary tuberculosis in children: a systematic review. Rev. Saude Publica 44, 735–742 10.1590/S0034-89102010005000019 20585739

[20] Stockdale A.J., Duke T., Graham S. and Kelly J. (2010) Evidence behind the WHO guidelines: hospital care for children: what is the diagnostic accuracy of gastric aspiration for the diagnosis of tuberculosis in children? J. Trop. Pediatr. 56, 291–298 10.1093/tropej/fmq081 20817689

[21] Piccini P., Chiappini E., Tortoli E., de Martino M. and Galli L. (2014) Clinical peculiarities of tuberculosis. BMC Infect. Dis. 14, S4 10.1186/1471-2334-14-S1-S4 24564419 PMC4015485

[22] Shingadia D. and Novelli V. (2003) Diagnosis and treatment of tuberculosis in children. Lancet Infect. Dis. 3, 624–632 10.1016/S1473-3099(03)00771-0 14522261

[23] Whiting P.F., Rutjes A.W., Westwood M.E., Mallett S., Deeks J.J., Reitsma J.B. et al. (2011) QUADAS-2: a revised tool for the quality assessment of diagnostic accuracy studies. Ann. Intern. Med. 155, 529–536 10.7326/0003-4819-155-8-201110180-00009 22007046

[24] Barnard D.A., Irusen E.M., Bruwer J.W., Plekker D., Whitelaw A.C., Deetlefs J.D. et al. (2015) The utility of Xpert MTB/RIF performed on bronchial washings obtained in patients with suspected pulmonary tuberculosis in a high prevalence setting. BMC Pulm. Med. 15, 103 10.1186/s12890-015-0086-z 26377395 PMC4573925

[25] Gowda N.C., Ray A., Soneja M., Khanna A. and Sinha S. (2018) Evaluation of Xpert((R))Mycobacterium tuberculosis/rifampin in sputum-smear negative and sputum-scarce patients with pulmonary tuberculosis using bronchoalveolar lavage fluid. Lung India 35, 295–300 29970767 10.4103/lungindia.lungindia_412_17 PMC6034363

[26] Jo Y.S., Park J.H., Lee J.K., Heo E.Y., Chung H.S. and Kim D.K. (2016) Discordance between MTB/RIF and real-time tuberculosis-specific polymerase chain reaction assay in bronchial washing specimen and its clinical implications. PloS one 11, e0164923 27760181 10.1371/journal.pone.0164923 PMC5070776

[27] Khalil K.F. and Butt T. (2015) Diagnostic yield of Bronchoalveolar Lavage gene Xpert in smear-negative and sputum-scarce pulmonary tuberculosis. J. Coll. Phys. Surg. Pak. 25, 115–118 25703755

[28] Kilaru S.C., Chenimilla N.P., Syed U., Momin K., Kilaru H., Patil E. et al. (2019) Role of Xpert MTB/RIF in Bronchoalveolar lavage fluid of sputum-scarce, suspected Pulmonary TB patients. J. Clin. Tuberc. Other Mycobact. Dis. 14, 7–11 10.1016/j.jctube.2018.11.003 31720410 PMC6830156

[29] Ko Y., Lee H.K., Lee Y.S., Kim M.Y., Shin J.H., Shim E.J. et al. (2016) Accuracy of Xpert((R)) MTB/RIF assay compared with AdvanSure TB/NTM real-time PCR using bronchoscopy specimens. Int. J. Tuberc. Lung Dis. 20, 115–120 10.5588/ijtld.15.0227 26688537

[30] Lee H.Y., Seong M.W., Park S.S., Hwang S.S., Lee J., Park Y.S. et al. (2013) Diagnostic accuracy of Xpert(R) MTB/RIF on bronchoscopy specimens in patients with suspected pulmonary tuberculosis. Int. J. Tuberc. Lung Dis. 17, 917–921 10.5588/ijtld.12.0885 23621953

[31] Lu Y., Zhu Y., Shen N., Tian L. and Sun Z. (2018) Evaluating the diagnostic accuracy of the Xpert MTB/RIF assay on bronchoalveolar lavage fluid: a retrospective study. Int. J. Infect. Dis. 71, 14–19 29428408 10.1016/j.ijid.2018.01.030

[32] Mok Y., Tan T.Y., Tay T.R., Wong H.S., Tiew P.Y., Kam J.W. et al. (2016) Do we need transbronchial lung biopsy if we have bronchoalveolar lavage Xpert(R) MTB/RIF? Int. J. Tuberc. Lung Dis. 20, 619–624 10.5588/ijtld.15.0463 27084815

[33] Pan X., Yang S., Deighton M.A., Qu Y., Hong L. and Su F. (2018) A comprehensive evaluation of Xpert MTB/RIF assay with bronchoalveolar lavage fluid as a single test or combined with conventional assays for diagnosis of pulmonary tuberculosis in China: a two-center prospective study. Front. Microbiol. 9, 444 10.3389/fmicb.2018.00444 29593688 PMC5859353

[34] Sanchez-Cabral O., Santillan-Diaz C., Paul Flores-Bello A., Ivannia Herrera-Ortega M., Luis Sandoval-Gutierrez J., Santillan-Doherty P. et al. (2020) GeneXpert (R) MTB/RIF assay with transbronchial lung cryobiopsy for Mycobacterium tuberculosis diagnosis. Ann. Transl. Med. 8, 351 10.21037/atm.2020.02.100 32355795 PMC7186622

[35] Sharma S.K., Kohli M., Yadav R.N., Chaubey J., Bhasin D., Sreenivas V. et al. (2015) Evaluating the diagnostic accuracy of Xpert MTB/RIF assay in pulmonary tuberculosis. PLoS ONE 10, e0141011 10.1371/journal.pone.0141011 26496123 PMC4619889

[36] Silva T.M.D., Soares V.M., Ramos M.G. and Santos A.D. (2019) Accuracy of a rapid molecular test for tuberculosis in sputum samples, bronchoalveolar lavage fluid, and tracheal aspirate obtained from patients with suspected pulmonary tuberculosis at a tertiary referral hospital. J. Bras. Pneumol. 45, e20170451 10.1590/1806-3713/e20170451 30864607 PMC6733743

[37] To K.W., Kam K.M., Chan D.P.C., Yip W.H., Chan K.P., Lo R. et al. (2018) Utility of GeneXpert in analysis of bronchoalveolar lavage samples from patients with suspected tuberculosis in an intermediate-burden setting. J. Infect. 77, 296–301 10.1016/j.jinf.2018.06.011 29964143

[38] Ullah I., Javaid A., Masud H., Ali M., Basit A., Ahmad W. et al. (2017) Rapid detection of Mycobacterium tuberculosis and rifampicin resistance in extrapulmonary tuberculosis and sputum smear-negative pulmonary suspects using Xpert MTB/RIF. J. Med. Microbiol. 66, 412–418 10.1099/jmm.0.000449 28425873

[39] Prakash A.K., Datta B., Tripathy J.P., Kumar N., Chatterjee P. and Jaiswal A. (2018) The clinical utility of cycle of threshold value of GeneXpert MTB/RIF (CBNAAT) and its diagnostic accuracy in pulmonary and extra-pulmonary samples at a tertiary care center in India. Indian J. Tuberc. 65, 296–302 10.1016/j.ijtb.2018.05.021 30522616

[40] Al-Darraji H.A., Abd Razak H., Ng K.P., Altice F.L. and Kamarulzaman A. (2013) The diagnostic performance of a single GeneXpert MTB/RIF assay in an intensified tuberculosis case finding survey among HIV-infected prisoners in Malaysia. PLoS ONE 8, e73717 10.1371/journal.pone.0073717 24040038 PMC3767617

[41] Boum Y. II, Kim S., Orikiriza P., Acuna-Villaorduna C., Vinhas S., Bonnet M. et al. (2016) Diagnostic accuracy of the small membrane filtration method for diagnosis of pulmonary tuberculosis in a high-HIV-prevalence setting. J. Clin. Microbiol. 54, 1520–1527 10.1128/JCM.00017-16 27030493 PMC4879284

[42] Friedrich S.O., Venter A., Kayigire X.A., Dawson R., Donald P.R. and Diacon A.H. (2011) Suitability of Xpert MTB/RIF and genotype MTBDRplus for patient selection for a tuberculosis clinical trial. J. Clin. Microbiol. 49, 2827–2831 10.1128/JCM.00138-11 21653771 PMC3147741

[43] Geleta D.A., Megerssa Y.C., Gudeta A.N., Akalu G.T., Debele M.T. and Tulu K.D. (2015) Xpert MTB/RIF assay for diagnosis of pulmonary tuberculosis in sputum specimens in remote health care facility. BMC Microbiol. 15, 220 10.1186/s12866-015-0566-6 26483194 PMC4615882

[44] LaCourse S.M., Cranmer L.M., Matemo D., Kinuthia J., Richardson B.A., John-Stewart G. et al. (2016) Tuberculosis case finding in HIV-infected pregnant women in Kenya reveals poor performance of symptom screening and rapid diagnostic tests. J. Acquir. Immune Defic. Syndr. 71, 219–227 10.1097/QAI.0000000000000826 26334736 PMC4712112

[45] Luetkemeyer A.F., Firnhaber C., Kendall M.A., Wu X., Mazurek G.H., Benator D.A. et al. (2016) Evaluation of Xpert MTB/RIF versus AFB smear and culture to identify pulmonary tuberculosis in patients with suspected tuberculosis from low and higher prevalence settings. Clin. Infect. Dis. 62, 1081–1088 10.1093/cid/ciw035 26839383 PMC4826450

[46] Madico G., Mpeirwe M., White L., Vinhas S., Orr B., Orikiriza P. et al. (2016) Detection and quantification of Mycobacterium tuberculosis in the sputum of culture-negative HIV-infected pulmonary tuberculosis suspects: a proof-of-concept study. PLoS ONE 11, e0158371 10.1371/journal.pone.0158371 27391604 PMC4938528

[47] Pinyopornpanish K., Chaiwarith R., Pantip C., Keawvichit R., Wongworapat K., Khamnoi P. et al. (2015) Comparison of Xpert MTB/RIF assay and the conventional sputum microscopy in detecting Mycobacterium tuberculosis in Northern Thailand. Tuberc. Res. Treat. 2015, 571782 26064681 10.1155/2015/571782 PMC4430669

[48] Rachow A., Zumla A., Heinrich N., Rojas-Ponce G., Mtafya B., Reither K. et al. (2011) Rapid and accurate detection of Mycobacterium tuberculosis in sputum samples by Cepheid Xpert MTB/RIF assay–a clinical validation study. PLoS ONE 6, e20458 10.1371/journal.pone.0020458 21738575 PMC3126807

[49] Rasheed W., Rao N.A., Adel H., Baig M.S. and Adil S.O. (2019) Diagnostic accuracy of Xpert MTB/RIF in sputum smear-negative pulmonary tuberculosis. Cureus 11, e5391 31620317 10.7759/cureus.5391 PMC6791393

[50] Rice J.P. and Seifert M. (2017) Performance of the Xpert MTB/RIF assay for the diagnosis of pulmonary tuberculosis and rifampin resistance in a low-incidence, high-resource setting. PLoS One 12, e0186139 29016684 10.1371/journal.pone.0186139 PMC5633176

[51] Sahle E.T., Blumenthal J., Jain S., Sun S., Young J., Manyazewal T. et al. (2019) Bacteriologically-confirmed pulmonary tuberculosis in an Ethiopian prison: prevalence from screening of entrant and resident prisoners. PLoS ONE 14, e0226160 31830092 10.1371/journal.pone.0226160 PMC6907752

[52] Yeong C., Byrne A.L., Cho J.-G., Sintchenko V., Crighton T. and Marais B.J. (2020) Use of GeneXpert MTB/RIF on a single pooled sputum specimen to exclude pulmonary tuberculosis among hospital inpatients placed in respiratory isolation. Int. J. Infect. Dis. 92, 175–180 10.1016/j.ijid.2019.12.024 31881274

[53] Acuna-Villaorduna C., Orikiriza P., Nyehangane D., White L.F., Mwanga-Amumpaire J., Kim S. et al. (2017) Effect of previous treatment and sputum quality on diagnostic accuracy of Xpert((R)) MTB/RIF. Int. J. Tuberc. Lung Dis. 21, 389–397 10.5588/ijtld.16.0785 28284253

[54] Mishra H., Reeve B.W.P., Palmer Z., Caldwell J., Dolby T., Naidoo C.C. et al. (2020) Xpert MTB/RIF Ultra and Xpert MTB/RIF for diagnosis of tuberculosis in an HIV-endemic setting with a high burden of previous tuberculosis: a two-cohort diagnostic accuracy study. Lancet Respir. Med. 8, 368–382 10.1016/S2213-2600(19)30370-4 32066534

[55] O’Grady J., Bates M., Chilukutu L., Mzyece J., Cheelo B., Chilufya M. et al. (2012) Evaluation of the Xpert MTB/RIF assay at a tertiary care referral hospital in a setting where tuberculosis and HIV infection are highly endemic. Clin. Infect. Dis. 55, 1171–1178 10.1093/cid/cis631 22806590

[56] Sohn H., Aero A.D., Menzies D., Behr M., Schwartzman K., Alvarez G.G. et al. (2014) Xpert MTB/RIF testing in a low tuberculosis incidence, high-resource setting: limitations in accuracy and clinical impact. Clin. Infect. Dis. 58, 970–976 10.1093/cid/ciu022 24429440

[57] Bates M., O’Grady J., Maeurer M., Tembo J., Chilukutu L., Chabala C. et al. (2013) Assessment of the Xpert MTB/RIF assay for diagnosis of tuberculosis with gastric lavage aspirates in children in sub-Saharan Africa: a prospective descriptive study. Lancet Infect. Dis. 13, 36–42 10.1016/S1473-3099(12)70245-1 23134697

[58] Reither K., Manyama C., Clowes P., Rachow A., Mapamba D., Steiner A. et al. (2015) Xpert MTB/RIF assay for diagnosis of pulmonary tuberculosis in children: a prospective, multi-centre evaluation. J. Infect. 70, 392–399 10.1016/j.jinf.2014.10.003 25312863

[59] Das A., Anupurba S., Mishra O.P., Banerjee T. and Tripathi R. (2019) Evaluation of Xpert MTB/RIF assay for diagnosis of tuberculosis in children. J. Trop. Pediatr. 65, 14–20 10.1093/tropej/fmy005 29438536

[60] Hasan Z., Shakoor S., Arif F., Mehnaz A., Akber A., Haider M. et al. (2017) Evaluation of Xpert MTB/RIF testing for rapid diagnosis of childhood pulmonary tuberculosis in children by Xpert MTB/RIF testing of stool samples in a low resource setting. BMC Res. Notes 10, 473 10.1186/s13104-017-2806-3 28886729 PMC5591572

[61] Myo K., Zaw M., Swe T.L., Kyaw Y.Y., Thwin T., Myo T.T. et al. (2018) Evaluation of Xpert (R) MTB/RIF assay as a diagnostic test for pulmonary tuberculosis in children in Myanmar. Int. J. Tuberc. Lung Dis. 22, 1051 10.5588/ijtld.18.0024 30092871

[62] Pang Y., Wang Y., Zhao S., Liu J., Zhao Y. and Li H. (2014) Evaluation of the Xpert MTB/RIF assay in gastric lavage aspirates for diagnosis of smear-negative childhood pulmonary tuberculosis. Pediatr. Infect. Dis. J. 33, 1047–1051 10.1097/INF.0000000000000403 25361186

[63] Sharma S., Shulania A., Achra A., Jeram H., Kansra S. and Duggal N. (2020) Diagnosis of pulmonary tuberculosis from gastric aspirate samples in nonexpectorating pediatric patients in a tertiary care hospital. Indian J. Pathol. Microbiol. 63, 210–213 32317517 10.4103/IJPM.IJPM_694_19

[64] LaCourse S.M., Chester F.M., Preidis G., McCrary L.M., Arscott-Mills T., Maliwichi M. et al. (2014) Use of Xpert for the diagnosis of pulmonary tuberculosis in severely malnourished hospitalized Malawian children. Pediatr. Infect. Dis. J. 33, 1200–1202 10.1097/INF.0000000000000384 25361410 PMC4217085

[65] Nicol M.P., Workman L., Isaacs W., Munro J., Black F., Eley B. et al. (2011) Accuracy of the Xpert MTB/RIF test for the diagnosis of pulmonary tuberculosis in children admitted to hospital in Cape Town, South Africa: a descriptive study. Lancet Infect. Dis. 11, 819–824 10.1016/S1473-3099(11)70167-0 21764384 PMC4202386

[66] Nicol M.P., Spiers K., Workman L., Isaacs W., Munro J., Black F. et al. (2013) Xpert MTB/RIF testing of stool samples for the diagnosis of pulmonary tuberculosis in children. Clin. Infect. Dis. 57, e18–e21 10.1093/cid/cit230 23580738 PMC3703104

[67] Nicol M.P., Workman L., Prins M., Bateman L., Ghebrekristos Y., Mbhele S. et al. (2018) Accuracy of Xpert Mtb/Rif ultra for the diagnosis of pulmonary tuberculosis in children. Pediatr. Infect. Dis. J. 37, E261–E263 10.1097/INF.0000000000001960 29474257

[68] Sekadde M.P., Wobudeya E., Joloba M.L., Ssengooba W., Kisembo H., Bakeera-Kitaka S. et al. (2013) Evaluation of the Xpert MTB/RIF test for the diagnosis of childhood pulmonary tuberculosis in Uganda: a cross-sectional diagnostic study. BMC Infect. Dis. 13, 133 10.1186/1471-2334-13-133 23497044 PMC3602671

[69] Togun T.O., Egere U., Sillah A.K., Ayorinde A., Mendy F., Tientcheu L. et al. (2015) Contribution of Xpert(R) MTB/RIF to the diagnosis of pulmonary tuberculosis among TB-exposed children in The Gambia. Int. J. Tuberc. Lung Dis. 19, 1091–1097, i-ii 10.5588/ijtld.15.0228 26260831

[70] Zar H.J., Workman L., Isaacs W., Munro J., Black F., Eley B. et al. (2012) Rapid molecular diagnosis of pulmonary tuberculosis in children using nasopharyngeal specimens. Clin. Infect. Dis. 55, 1088–1095 10.1093/cid/cis598 22752518 PMC3529610

[71] Zar H.J., Workman L., Isaacs W., Dheda K., Zemanay W. and Nicol M.P. (2013) Rapid diagnosis of pulmonary tuberculosis in African children in a primary care setting by use of Xpert MTB/RIF on respiratory specimens: a prospective study. Lancet Global Health 1, e97–e104 10.1016/S2214-109X(13)70036-6 25104164

[72] Jo Y.S., Park J.-H., Lee J.K., Heo E.Y., Chung H.S. and Kim D.K. (2016) Discordance between MTB/RIF and real-time tuberculosis-specific polymerase chain reaction assay in bronchial washing specimen and its clinical implications. PLoS ONE 11, e0164923 10.1371/journal.pone.0164923 27760181 PMC5070776

[73] Steingart K.R., Ng V., Henry M., Hopewell P.C., Ramsay A., Cunningham J. et al. (2006) Sputum processing methods to improve the sensitivity of smear microscopy for tuberculosis: a systematic review. Lancet Infect. Dis. 6, 664–674 10.1016/S1473-3099(06)70602-8 17008175

[74] Hoft D.F., Brown R.M. and Belshe R.B. (2000) Mucosal bacille calmette-Guerin vaccination of humans inhibits delayed-type hypersensitivity to purified protein derivative but induces mycobacteria-specific interferon-gamma responses. Clin. Infect. Dis. 30, S217–S222 10.1086/313864 10875787

[75] Albert H., Nathavitharana R.R., Isaacs C., Pai M., Denkinger C.M. and Boehme C.C. (2016) Development, roll-out and impact of Xpert MTB/RIF for tuberculosis: what lessons have we learnt and how can we do better? Eur. Respir. J. 48, 516–525 10.1183/13993003.00543-2016 27418550 PMC4967565

[76] Dorman S.E., Schumacher S.G., Alland D., Nabeta P., Armstrong D.T., King B. et al. (2018) Xpert MTB/RIF Ultra for detection of Mycobacterium tuberculosis and rifampicin resistance: a prospective multicentre diagnostic accuracy study. Lancet Infect. Dis. 18, 76–84 10.1016/S1473-3099(17)30691-6 29198911 PMC6168783

[77] Detjen A.K., DiNardo A.R., Leyden J., Steingart K.R., Menzies D., Schiller I. et al. (2015) Xpert MTB/RIF assay for the diagnosis of pulmonary tuberculosis in children: a systematic review and meta-analysis. Lancet Respir. Med. 3, 451–461 10.1016/S2213-2600(15)00095-8 25812968 PMC4756280

[78] Chakravorty S., Simmons A.M., Rowneki M., Parmar H., Cao Y., Ryan J. et al. (2017) The New Xpert MTB/RIF Ultra: improving detection of Mycobacterium tuberculosis and resistance to rifampin in an assay suitable for point-of-care testing. mBio 8, e00812–17 10.1128/mBio.00812-17 28851844 PMC5574709

[79] Osei Sekyere J., Maphalala N., Malinga L.A., Mbelle N.M. and Maningi N.E. (2019) A comparative evaluation of the new genexpert MTB/RIF ultra and other rapid diagnostic assays for detecting tuberculosis in pulmonary and extra pulmonary specimens. Sci. Rep. 9, 16587 10.1038/s41598-019-53086-5 31719625 PMC6851384

[80] Meldau R., Randall P., Pooran A., Limberis J., Makambwa E., Dhansay M. et al. (2019) Same-day tools, including Xpert Ultra and IRISA-TB, for rapid diagnosis of pleural tuberculosis: a prospective observational study. J. Clin. Microbiol. 57, 10.1128/JCM.00614-19 31270183 PMC6711909

[81] Vashistha R., Somani K., Patel A., Maheshwari M. and Datey S. (2018) Clinical profile of abdominal tuberculosis in a tertiary care centre of central india-a descriptive observational study. J. Evol. Med. Dental Sci. 7, 3699–3702 10.14260/jemds/2018/830

[82] Briceno R.K., Sergent S.R., Benites S.M. and Alocilja E.C. (2019) Nanoparticle-based biosensing assay for universally accessible low-cost TB detection with comparable sensitivity as culture. Diagnostics 9, 222 10.3390/diagnostics9040222 31847171 PMC6963232

[83] Blau H., Linnane B., Carzino R., Tannenbaum E.L., Skoric B., Robinson P.J. et al. (2014) Induced sputum compared to bronchoalveolar lavage in young, non-expectorating cystic fibrosis children. J. Cyst. Fibros. 13, 106–110 10.1016/j.jcf.2013.05.013 23806622

[84] Garcia B.J., Loxton A.G., Dolganov G.M., Van T.T., Davis J.L., de Jong B.C. et al. (2016) Sputum is a surrogate for bronchoalveolar lavage for monitoring *Mycobacterium tuberculosis* transcriptional profiles in TB patients. Tuberculosis 100, 89–94 10.1016/j.tube.2016.07.004 27553415 PMC4999252

[85] Conde M.B., Soares S.L., Mello F.C., Rezende V.M., Almeida L.L., Reingold A.L. et al. (2000) Comparison of sputum induction with fiberoptic bronchoscopy in the diagnosis of tuberculosis: experience at an acquired immune deficiency syndrome reference center in Rio de Janeiro, Brazil. Am. J. Respir. Crit. Care Med. 162, 2238–2240 10.1164/ajrccm.162.6.2003125 11112145

[86] Ruiz Jimenez M., Guillen Martin S., Prieto Tato L.M., Cacho Calvo J.B., Alvarez Garcia A., Soto Sanchez B. et al. (2013) Induced sputum versus gastric lavage for the diagnosis of pulmonary tuberculosis in children. BMC Infect. Dis. 13, 222 10.1186/1471-2334-13-222 23679059 PMC3688294

[87] Zar H.J., Hanslo D., Apolles P., Swingler G. and Hussey G. (2005) Induced sputum versus gastric lavage for microbiological confirmation of pulmonary tuberculosis in infants and young children: a prospective study. Lancet 365, 130–134 10.1016/S0140-6736(05)17702-2 15639294

[88] Parashar D., Kabra S.K., Lodha R., Singh V., Mukherjee A., Arya T. et al. (2013) Does neutralization of gastric aspirates from children with suspected intrathoracic tuberculosis affect mycobacterial yields on MGIT culture? J. Clin. Microbiol. 51, 1753–1756 10.1128/JCM.00202-13 23536406 PMC3716107

[89] Mathew J.L., Vijayasekharan D. and Singh S. (2014) Is Xpert MTB/RIF assay in gastric lavage aspirate useful for diagnosis of smear-negative childhood pulmonary tuberculosis? Indian Pediatr. 51, 1007–1011 10.1007/s13312-014-0548-z 25560161

[90] Ramirez P., Valencia M. and Torres A. (2007) Bronchoalveolar lavage to diagnose respiratory infections. Semin Respir. Crit. Care Med. 28, 525–533 10.1055/s-2007-991524 17975780

[91] Zhang G.P., Chen Q., Liu J.M., Zhou S.P., Yu X.J., Lu J. et al. (2012) Application of bacterial cultures of bronchoalveolar lavage fluids in children with pulmonary infection. Zhongguo Dang Dai Er Ke Za Zhi 14, 350–352 22613105

[92] Singer-Leshinsky S. (2016) Pulmonary tuberculosis: improving diagnosis and management. JAAPA 29, 20–25 10.1097/01.JAA.0000476207.96819.a7 26757063

[93] Connell T.G., Zar H.J. and Nicol M.P. (2011) Advances in the diagnosis of pulmonary tuberculosis in HIV-infected and HIV-uninfected children. J. Infect. Dis. 204, S1151–S1158 10.1093/infdis/jir413 21996697 PMC3192545

