# Supplementary material for: Which sample type is better for Xpert MTB/RIF to diagnose adult and pediatric pulmonary tuberculosis?
Source: Biosci Rep. 2020 Aug 4;40(8):BSR20200308. doi: 10.1042/BSR20200308 (PMC7403955; doi:10.1042/BSR20200308)
Supplement: Supplementary Table S1 [file BSR-2020-0308_supp.pdf]

Supplementary Table 1 The results of subgroup analysis

Subgroup	No. studies	Chi-squared	P value	Sensitivity (95% CI [a])	Specificity (95% CI)
**Adults BAL [b] group**					
Smear status					
Smear-positive	3	0.10	0.950	95% (0.85-0.99)	30% (0.02-0.79)
Smear-negative	4	5.21	0.157	75% (0.67-0.82)	92% (0.90-0.94)
HIV status					
HIV-positive	3	0.10	0.950	79% (0.66-0.89)	88% (0.79-0.94)
Specimen procession					
Yes	10	29.21	0.001	86% (0.83-0.90)	88% (0.86-0.90)
**Adults ES [c] group**					
Smear status					
Smear-positive	3	2.02	0.365	97% (0.92-0.99)	25% (0.08-0.51)
Smear-negative	4	7.88	0.049	80% (0.74-0.86)	100% (0.98-1.00)
HIV status					
HIV-positive	2	2.18	0.140	92% (0.86-0.96)	81% (0.71-0.89)
HIV-negative	2	0.26	0.878	93% (0.87-0.97)	96% (0.93-0.98)
Specimen procession					
Yes	10	47.33	0	90% (0.88-0.92)	98% (0.98-1.00)
**Adults IS [d] group**					
Smear status					
Smear-positive	4	0.75	0.861	98% (0.60-0.99)	71% (0.50-0.88)
Smear-negative	4	16.59	0.001	67% (0.60-0.73)	95% (0.94-0.96)
HIV status					
HIV-positive	3	10.86	0.004	88% (0.84-0.91)	96% (0.94-0.97)
HIV-negative	3	4.32	0.115	88% (0.82-0.92)	95% (0.93-0.97)
Specimen procession					
Yes	7	18.95	0.004	86% (0.84-0.89)	97% (0.96-0.98)
**Children GA [e] group**					
Smear status					
Smear- negative	3	37.89	0.000	71% (0.59-0.81)	94% (0.93-0.95)
Specimen procession					
Yes	5	45.29	0.000	79% (0.71-0.86)	94% (0.93-0.94)
**Children IS group**					
Smear status					
Smear-positive	4	0.37	0.946	96% (0.89-0.99)	56% (0.11-0.94)
Smear-negative	5	3.91	0.419	65% (0.58-0.72)	98% (0.97-0.99)
HIV status					
HIV-positive	4	7.08	0.07	88% (0.74-0.96)	98% (0.96-0.99)
HIV-negative	4	0.87	0.833	69% (0.61-0.77)	98% (0.97-0.99)
Specimen procession					
Yes	8	6.83	0.446	67% (0.61-0.72)	99% (0.98-0.99)

a: confidence interval; b: bronchoalveolar lavage; c: expectorated sputum; d: induced sputum; e: gastric aspiration
